# Single-visit endodontic treatment of mature teeth with chronic apical abscesses using mineral trioxide aggregate cement: a randomized clinical trial

**DOI:** 10.1186/s12903-016-0276-y

**Published:** 2016-08-23

**Authors:** Reem Siraj Alsulaimani

**Affiliations:** Lecturer in the Department of Restorative Dental Science, College of Dentistry, King Saud University, P.O. Box 60169, Riyadh, 11545 Kingdom of Saudi Arabia

**Keywords:** Single visit, Periapical lesion, Mineral trioxide aggregate, Root canal treatment

## Abstract

**Background:**

Mature teeth with chronic apical abscesses characterized by intermittent discharge of pus through an associated sinus tract. This communication between oral mucosa and periapical inflammation is challenging for the sealing ability of root canal obturation material. Therefore, the study aim was to compare the outcomes of endodontic treatment using mineral trioxide aggregate (MTA) cement to the conventional gutta-percha cone and root canal sealer as an obturation material in mature teeth with chronic apical abscesses.

**Methods:**

Mature teeth with chronic apical abscesses referred to our clinic for root canal treatment between 2010 and 2012 were treated in a single visit and distributed among treatment (T) and control (C) groups using a predetermined randomization block (TCTC). After chemo-mechanical preparation, teeth in group T received MTA cement mixed in a 0.26 water to powder ratio, and teeth group C received gutta-percha and root canal sealer using the warm vertical technique. The treatment outcomes were defined as obturation length, periapical healing, resorption of extruded material, and survival rate at least 2.5 years after treatment. Three endodontists blinded to the type of obturation material documented treatment outcomes. Statistical analysis at *P* < 0.05 was conducted to measure difference between the groups.

**Results:**

Thirty-six teeth were treated between 2010 and 2012, and 32 teeth were evaluated in 2015. Complete periapical healing was observed in 87.5 % of MTA-treated teeth and 75.0 % of gutta-percha-treated teeth. Adequate obturation length was reported in 50.0 % of MTA-treated and 37.5 % of gutta-percha-treated teeth. Complete resorption of extruded material was evident in 83.3 % MTA-treated teeth and 100.0 % gutta-percha-treated teeth. The survival rate of MTA-treated teeth was 100 % at 3, and 5 years, while the survival rate of gutta-percha-treated teeth was 83.3 % at 3, and 5 years. There was no significant difference between the groups in term of periapical healing, survival rate, obturation length, or resorption of extruded material.

**Conclusions:**

The outcomes of single-visit endodontic treatment of mature teeth with chronic apical abscesses using MTA cement were better, but not statistically significant, compared to conventional treatment.

**Trial registration:**

ISRCTN15285974. Registered retrospectively 23 June 2015.

**Electronic supplementary material:**

The online version of this article (doi:10.1186/s12903-016-0276-y) contains supplementary material, which is available to authorized users.

## Background

A chronic apical abscess is a long-standing periapical inflammation characterized by intermittent discharge of pus through an intraoral sinus tract, with radiolucent signs of periapical osseous destruction [1]. Several studies reported that the prevalence of mature teeth with chronic apical abscesses ranges between 9.7 % and 18.1 % [2, 3]. In general, the presence of direct communication between the oral mucosa and periapical inflammation is challenging for the sealing ability of root canal obturation material.

Special treatment of chronic apical abscesses such as, sinus tract cauterization or surgical apicoectomy have been advocated by earlier report [4, 5], while others have described the complete healing of sinus tracts with nonsurgical endodontic therapy [6, 7]. The literature shows scarce case reports on the treatment of mature teeth with chronic apical abscesses. These reported treatments have ranged from multiple-visit treatment with intra-canal medicament to single visit treatment [8–10], without special consideration to the communication present between the oral mucosa and periapical inflammation.

After chemo mechanical preparation, the primary goal of root canal obturation is to prevent the spread of bacteria and bacterial toxins from the canals into the periapical tissues [11]. Furthermore, since obturation material comes into contact with periodontal tissue, it should not interfere with periapical tissue healing; preferably, it should stimulate periapical tissue regeneration [12]. The conventional root canal obturation material consist of gutta-percha cones and root canal sealer. The biocompatibility and sealing ability of gutta-percha cones and root canal sealer was reported to be less than Mineral Trioxide Aggregate (MTA) cement [13, 14]. Moreover, teeth with significant periapical pathosis, such as chronic apical abscesses, are future candidates for surgical intervention, and Mineral trioxide aggregate (MTA) cement is the recommended retrograde filling material [15].

Therefore, the study aim was to compare the outcomes of endodontic treatment using mineral trioxide aggregate (MTA) cement to the conventional gutta-percha cone and root canal sealer as an obturation material in mature teeth with chronic apical abscesses.

## Methods

### Study population

**Fig. 1:**
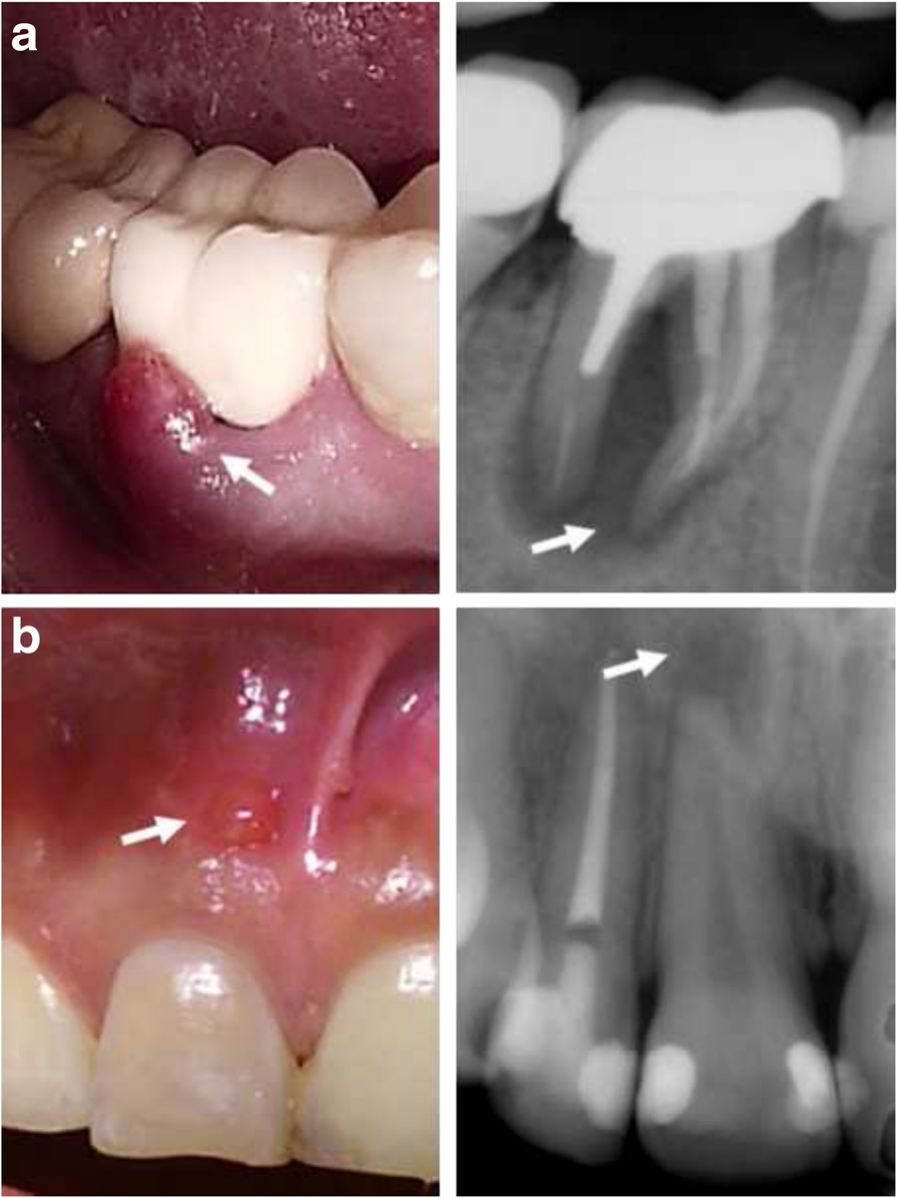
Clinical and radiographical presentation of mature teeth with chronic apical abscesses. **a** Non-restorable tooth with sinus tract and periapical radiolucency (*arrow*) excluded from the study. **b** Restorable tooth with sinus tract and periapical radiolucency (*arrow*) included in the trial

**Fig. 2:**
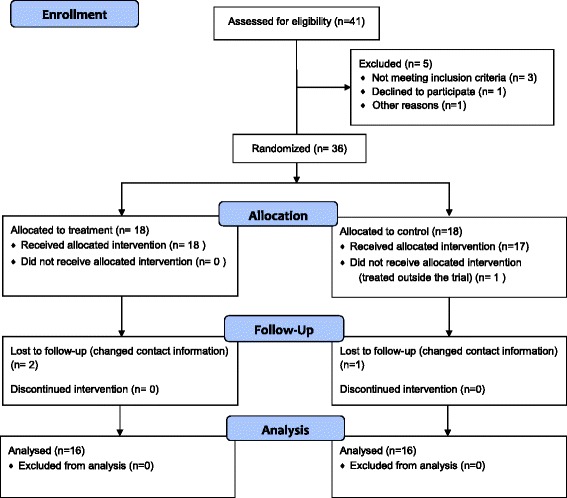
CONSORT flowchart of patients enrolled in the study

Those patients referred to the endodontic clinics at Girls University Campus, College of Dentistry, KSU between 2010 and 2012 were screened for teeth diagnosed with chronic apical abscesses. For this study, the inclusion criteria included restorable teeth with closed apices, visible sinus tracts, and periapical radiolucency with diameters larger than 3 mm (Fig. 1b). Forty-one teeth were screened, and five were rejected because they did not satisfy the inclusion criteria (Fig. 2).

In total, 36 teeth were enrolled in this trial. A sample size of at least 16 teeth in each study group was determined to be sufficient to detect an assumed clinically significant difference of 43 % in periapical healing between the treatment (T) group using MTA cement and the control (C) group using gutta-percha cone and root canal sealer. This study adopted a two-tailed chi-squared test with 90 % power and a 5 % level of significance.

### Study design and treatment materials

The included teeth were allocated among T and C groups using a 1:1 ratio in a parallel design, and the clinical staff assigned teeth using a predetermined randomized block (TCTC). Teeth with history of root canal treatment were labeled as secondary treatment and distributed evenly among the T and C groups. Moreover, the patients were blinded to the type of obturation material.

The single-visit endodontic treatment was performed under local anesthesia using rubber dam isolation and 3.5× magnification. The coronal access cavity was prepared and, when required, the previous root canal filling was dissolved using chloroform, then, the radicular space was cleaned and shaped using RaCe rotary NiTi files with 0.04 and 0.06 tapers (FKG Dentair, La Chaux-de-Fonds, Switzerland). Irrigation protocol consisted of 5 ml of 5.25 % sodium hypochlorite (NaOCl) during root canal shaping, and saline prior to root canal obturation. The working length was determined using a Root ZX apex locator (J. Morita Mfg. Corp., Kyoto, Japan). Canal shaping was considered to be adequate when the apical preparation was at least 0.35 mm for primary treatments and 0.50 mm for teeth with secondary treatments.

**Fig. 3:**
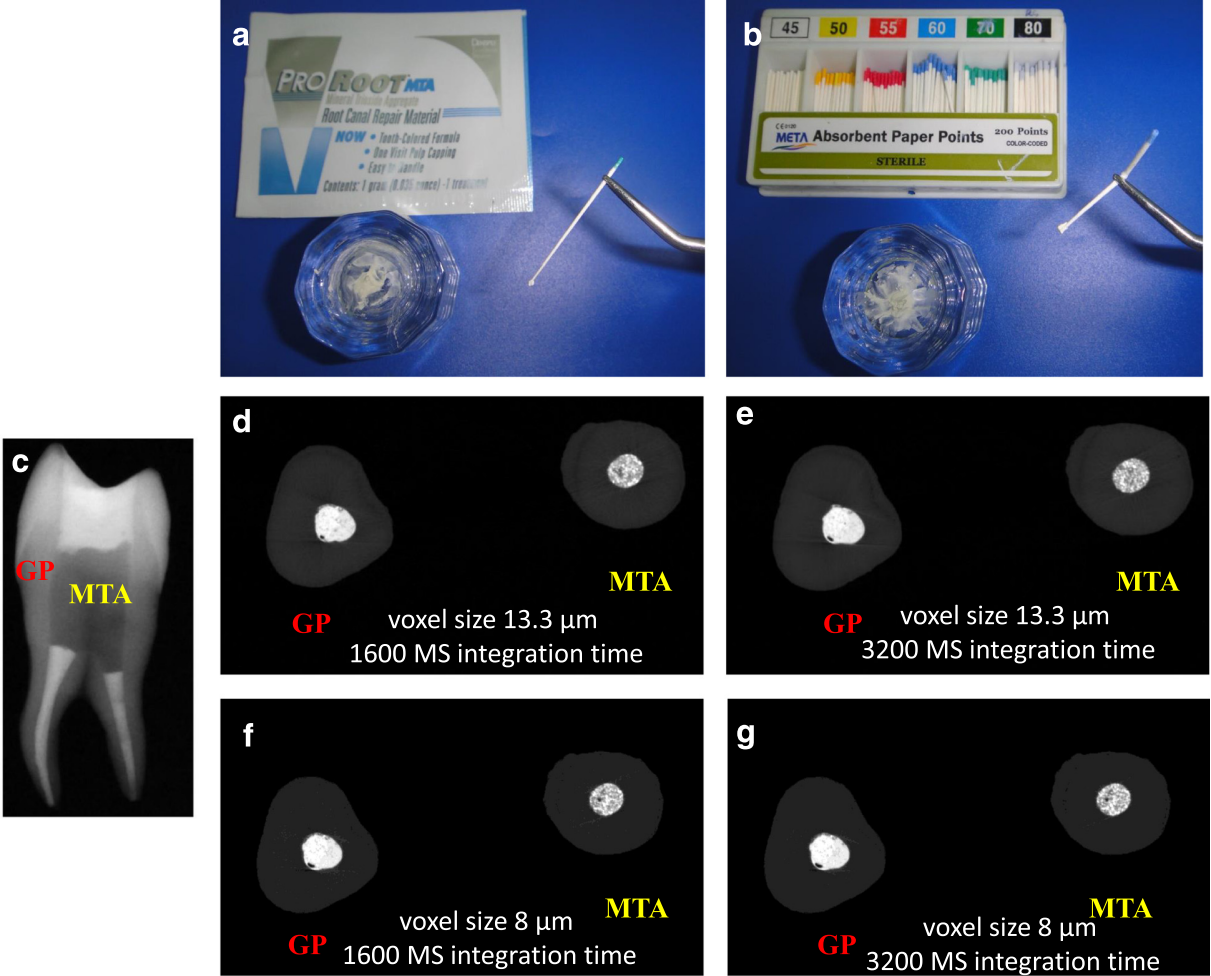
MTA cement obturation. **a** White ProRoot powder is mixed with sterile water, and small increment was attached to an ISO #35 paper point held with a cotton plier. **b** A larger MTA increment was attached to an ISO #60 paper point while the paper point was bent to facilitate compacting MTA cement apically in posterior teeth. **c** Periapical radiograph of a two rooted premolar. One root canal was filled with gutta-percha and root canal sealer and the other root was filled with MTA cement as described in the methods section. **d**, **e**, **f**, **g** Micro-CT analysis to evaluate the obturation density of both techniques. Voids were difficult to detect in MTA obturation even at high scanning parameters

The teeth in group T were filled with 4 mm of White ProRoot MTA® (Dentsply International) mixed with sterile water in a 0.26 WP ratio. Then, a small amount of MTA cement was applied using a dry ISO-standardized paper point (Meta Dental Corp., Chungbuk, Korea; Fig. 3a). The size of the paper point was chosen to coincide with the size of the apical preparation to compact the MTA cement at the root canal apex. In addition, the sizes of the paper points were increased (Fig. 3b) with an increasing coronal taper of the canal, and each increment used new, dry paper points until the planned obturation length was achieved.

The teeth in group C were filled with 4 mm of gutta-percha and root canal sealer using warm vertical compaction. Briefly explained, the apical third was filled by down-backing a 0.04 taper gutta-percha cone with an apical diameter coinciding with the diameter of the prepared canal and coated with root canal sealer (Tubliseal Xpress canal sealant, SybronEndo. Orange, CA, USA). The micro-CT analysis to evaluate the resulting obturation densities of both techniques are presented in Fig. 3c-g.

The remaining radicular space in all teeth was either left untouched for post cementation or filled with thermoplasticized gutta-percha (Obtura®, SybronEndo, Orange, CA, USA), depending on the restorative plan of each tooth. Finally, the access cavity was sealed using resin-modified glass ionomer and conventional periapical radiographs were obtained after treatment completion. Every patient was referred to a restorative specialist to receive a final restorative treatment after 2 weeks.

### Treatment outcomes

Periapical healing was the primary outcome and evaluated by periapical radiographs at least 2.5 years after the operation. Complete periapical healing was defined as a uniform width of the periodontal ligament space, no obvious breakdown of the lamina dura, and well-organized bone trabeculae [16]. The obturation length was measured using immediate postoperative radiographs, and an adequate obturation length was recognized when the obturation material ended less than 1 mm from the radiographic apex. Inadequate obturation was determined when the obturation ended more than 1 mm from the radiographic apex, and overfilling was defined as the root canal filling material extruding by more than 1 mm from the radiographic apex [16]. The resorption of extruded obturation material was evaluated via radiographs at follow-up visits. Overall, the obturation length and resorption of extruded material were secondary outcomes. The survival of an endodontically treated tooth was defined as its continued presence and painless function at follow-up visits [17]. To standardize the evaluations, the treatment outcomes of MTA-treated teeth were compared to gutta-percha-teeth treated during the same period of time (randomization block) during follow up visits.

Three endodontists blinded to the type of obturation material evaluated the complete periapical healing, obturation length, and resorption of the extruded material independently. In cases of disagreement, the final evaluation was based on the evaluation of two agreeable examiners.

### Statistical analysis

The number and percentage of teeth with complete periapical healing, adequate obturation length, and completely resorbed extruded material were compared and analyzed using the chi-squared or Fisher’s exact test based on the size of the data in each or total row using two-sided statistical tests and SPSS version 22.0.0. The overall survival rate was calculated at follow-up visits. In addition, the survival rate was analyzed by the history of root canal treatment using a univariate analysis and Fisher’s exact test to detect significant difference between MTA-treated and gutta-percha-treated teeth. In all cases, *P* values less than 0.05 were considered to represent a statistically significant difference between the study groups.

#### Null hypothesis (H0)

There will be no statistically significant difference in the number of teeth with complete periapical healing after treatment with MTA obturation, when compared with teeth treated with conventional gutta-percha cone and root canal sealer.

#### Alternative hypothesis (H1)

There will be a statistically significant difference in the number of teeth with complete periapical healing after treatment with MTA obturation compared with teeth treated with conventional gutta-percha cone and root canal sealer.

## Results

Thirty-six mature teeth with chronic apical abscesses (between 2010 and 2012) were included in this study. The participants included healthy females, and their age ranging between 21 and 41 years. Thirty-two treated teeth (Table 1) were evaluated at 2015, and the average time of follow-up was 3.6 years, with an equal distribution between study groups (Table 2).

**Table 1:** Summary of study sample (*n* = 32)

Groups	MTA cement obturation	Gutta-percha and root canal sealer obturation
N	16	16
Patients mean age in years with SD	29.81 ± 7.93	32.69 ± 8.51
Primary treatment	8 (50 %)	8 (50 %)
Secondary treatment	8 (50 %)	8 (50 %)
Maxillary teeth	14 (87.5 %)	11 (68.75 %)
Mandibular teeth	2 (12.5 %)	5 (31.25 %)
Composite build up	05 (31.25 %)	07(43.75 %)
Crown	05 (31.25 %)	05 (31.25 %)
Crown with post and core build up	06 (37.5 %)	04 (25 %)
Survival rate	16 (100 %)	14 (87.5 %)

*SD* standard deviation

**Table 2:** Average time of follow up observed among study groups

Obturation material	Mineral Trioxide Aggregate cement	Gutta-percha cone and root canal sealer
	(*N* = 16)	(*N* = 16)
Follow up time (in year) Mean(SD)	3.6 (1.1)	3.6 (1.1)
Follow up time	Number of teeth	
5 years	6	6
3 years	6	6
2.5 years	4	4

Adequate obturation lengths were evident in eight MTA-treated teeth (50.0 %) and 6 gutta-percha-treated teeth (37.5 %) with no significant difference between the groups (*P* = 0.72). Complete resorption of the extruded material was evident in five out of six (83.3 %) MTA-treated teeth and in eight out of eight (100.0 %) gutta-percha-treated teeth with no significant difference between the group (*P* = 0.42).

**Fig. 4:**
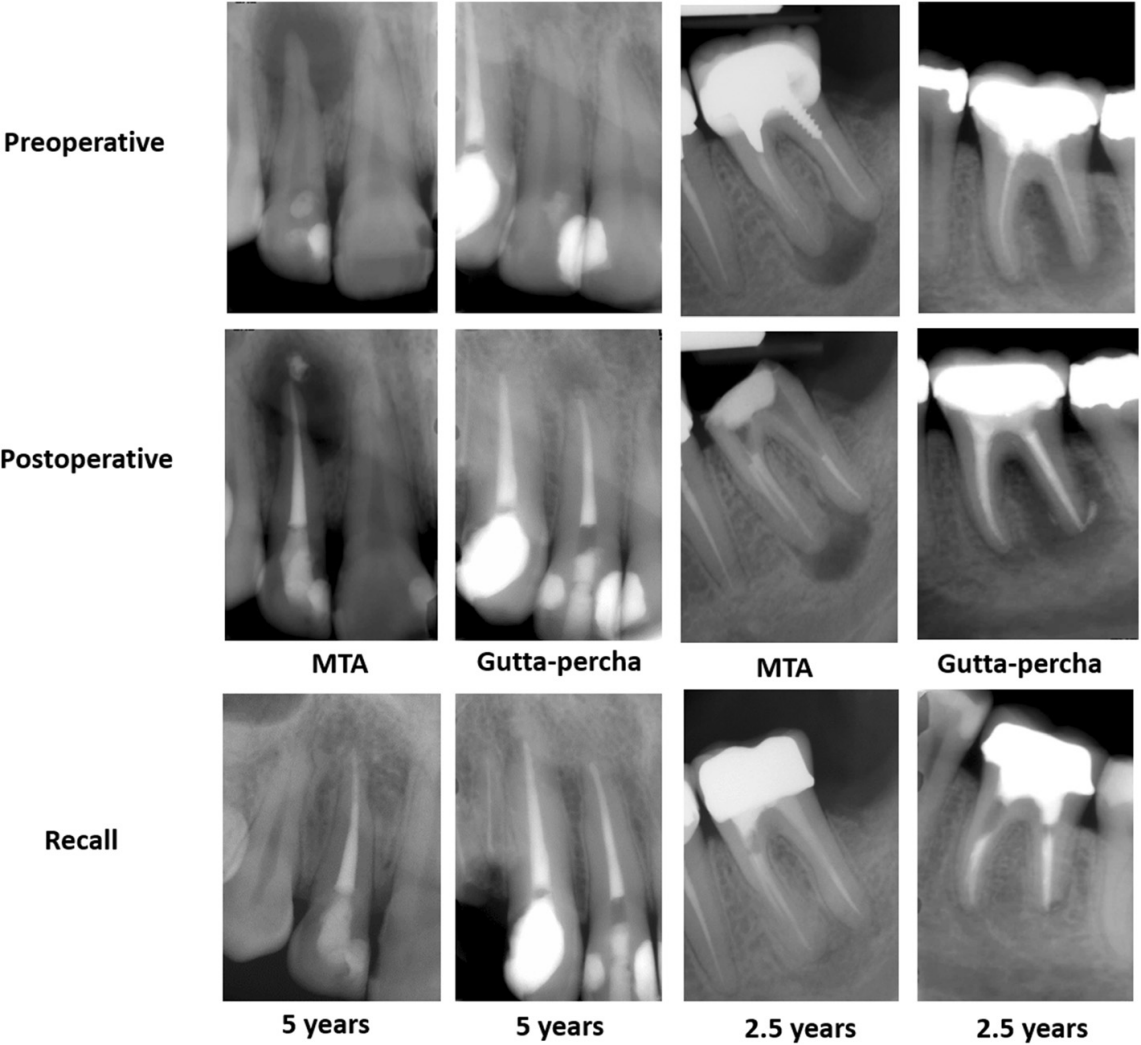
Periapical radiographs of primary treatment in maxillary lateral incisors and secondary treatment in mandibular first molars followed for 5- and 2.5-years

**Fig. 5:**
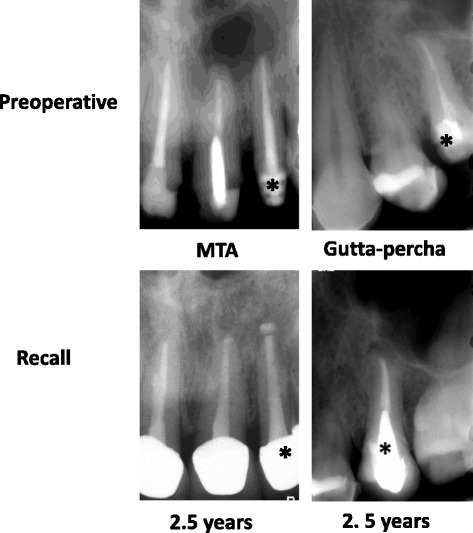
Periapical radiographs of MTA- and gutta-percha-treated teeth (*) with incomplete periapical healing at 2.5-years follow-up visits

Complete periapical healing (Fig. 4) was evident in 14 out of 16 MTA-treated teeth (87.5 %) and in 12 out of 16 gutta-percha-treated teeth (75.0 %) with no statistical significant difference between the groups (*P* = 0.69). Incomplete periapical healing (Fig. 5) was observed in two out of 16 MTA-treated teeth and in two out of 16 gutta-percha-treated teeth with no statistical significant difference between the groups (*P* = 0.69). Therefore, the null hypothesis was accepted.

None of MTA-treated teeth (*n* = 16) required surgical intervention or extracted at the follow-up visits; therefore, the survival rate of MTA-treated teeth was 100 % at 2.5, 3, and 5 years. One gutta-percha-treated tooth was extracted at 3 years’ follow-up visit, and another tooth at 5 years (Additional file 1). Therefore, the survival rate of gutta-percha-treated teeth was 100 % at 2.5 years but 83.3 % at 3, and 5 years. There was no statistical significant difference between the groups at 2.5, 3, and 5 years (*P* = 1, 0.75, 0.75 respectively).

### Primary treatment vs. secondary treatment

Table 3 demonstrates equal distribution of the primary treatments and secondary treatments among study groups. The secondary treatments for teeth with histories of root canal treatment had no significant effect on obturation length, resorption of the extruded material, or periapical healing after root canal obturation using MTA cement or gutta-percha cone and root canal sealer. At the 2.5, 3, and 5-years intervals after the treatment, there was no statistically significant difference in tooth survival between MTA-treated and gutta-percha treated teeth, when analyzed by the history of root canal treatment (*P* = 0.43).

**Table 3:** Primary treatment vs. Secondary treatment

Parameters	MTA cement obturation	Gutta-percha cone and root canal sealer obturation	*P* value
Primary treatment	*n* = 8	*n* = 8	
Complete periapical healing	08 (100 %)	06 (75 %)	1.00
Incomplete periapical healing	00	00	
Adequate Obturation length	06 (75 %)	04 (50 %)	0.61
Inadequate obturation length	02 (25 %)	04 (50 %)	
Complete resorption of extruded material	02 (25 %)	02 (25 %)	1.00
Incomplete resorption of extruded material	00	00	
Secondary treatment	*n* = 8	*n* = 8	
Complete periapical healing	6 (75 %)	6 (75 %)	1.00
Incomplete periapical healing	02 (25 %)	02 (25 %)	
Adequate Obturation length	2 (25 %)	2 (25 %)	1.00
Inadequate obturation length	6 (75 %)	6 (75 %)	
Complete resorption of extruded material	3 (37.5 %)	6 (75 %)	0.40
Incomplete resorption of extruded material	1 (12.5 %)	0	

## Discussion

This study is part of a randomized, double blinded, controlled trial registered as ISRCTN15285974 (http://www.isrctn.com), with the title “Mineral trioxide aggregate (MTA) as an alternative root canal filling material”. This part of the study targeted mature teeth with chronic apical abscesses because the direct communication between the periapical inflammation and oral mucosa poses challenges for the sealing ability of root canal obturation material.

Moreover, the literature does not include updated information about the management of such teeth [6, 7, 18, 19], or a previous clinical trial targeting treatment of mature teeth with chronic apical abscesses. Recent publications have mainly been case reports treating mature teeth with chronic apical abscesses in multiple-visits with intra-canal medicament [9], or with the use of chlorhexidine irrigant [8], and the follow-up time was less than 2 years. Therefore, the treatment outcomes of this study cannot be compared directly to previous publications.

The practice of single-visit treatment have gained popularity due to its predictable success in term of periapical healing [20, 21], and satisfying patient preference [22]. In this study, complete periapical healing was observed in 87.5 % in MTA-treated, and 75 % in gutta-percha-treated after single-visit endodontic treatment, which is comparable to pervious reports [21]. At 5-years, the survival rate of MTA-treated and gutta-percha treated teeth collectively was 91.6 %, which is equivalent to 92 % survival rate reported in 117818 endodontically treated teeth followed for 5 years retrospectively [23].

Treated teeth were examined 2-weeks postoperatively to evaluate sinus tract healing. None of the treated teeth had a sinus tract at 2-weeks follow up. Therefore, patients were referred to a restorative specialist to receive final restorative treatment. Two gutta-percha-treated teeth were extracted due to non-restorable coronal fracture within 6 months of root canal treatment, because patients’ compliance to attend restorative appointment after root canal treatment was low. Therefore, educating patients about the importance of final restorative treatment is important to maintain the functionality of endodontically treated teeth.

The primary components of MTA powder include dicalcium silicate, tricalcium silicate, and tricalcium aluminate [24]. The radiopacity of white MTA is 6.74 mm Al [25], which is adequate according to International Organization for Standardization (ISO) requirements. In addition, retreatment of MTA-filled canals after setting is possible [26]; however, the clinical manipulation of MTA cement is difficult and there is no designated instrument for orthograde obturation. In addition, root discoloration and incomplete removal of MTA cement during retreatment was reported [26]. Overall, MTA cement exhibits a superior sealing ability and biocompatibility compared to the conventional gutta-percha cones and root canal sealer [13], and an alternative root canal filling for latex-allergic patients [27].

Several attempts were proposed to facilitate MTA cement obturation in orthograde setting, including the use of Lee MTA pellet-forming block and a plugger [28, 29], ultrasonic activation [30], K-files with an apex locater as in the Lawaty technique [31] and a customized plugger for individual cases [32]. Transferring MTA cement from the mixing container into the apical third of the tooth is challenging. Because the available MTA carrier (e.g., the Dovgan MTA carrier) have a large diameter (0.8–1.6 mm) that is not amenable to orthograde filling, and made of metal, therefore, the MTA cement falls out before it can be packed into the root canal. Therefore, orthograde application of MTA cement requires an instrument that coincides with the shape of the prepared root canal to facilitate transfer and compaction of MTA cement apically. Currently, such an instrument is not available.

In this study, MTA cement was mixed with a WP ratio of 0.26 as recommended by Fridland and Rosado in 2003 [33], to facilitate clinical manipulation of MTA cement. Absorbent paper points were used to transfer and pack MTA cement apically. The paper points provided the closest match to an instrument to which MTA cement attached easily and the shapes coincides with the shape of the prepared root canal. The described technique (Fig. 3) produced a radiographically acceptable obturation in MTA-treated teeth (Additional file 1); Therefore, developing MTA-carrier with the criteria noted above will facilitate MTA obturation in mature teeth.

Root canal cleaning and shaping were performed using 0.04 and 0.06 taper NiTi files, which reduced the time required for root canal preparation. In the same vein, 5.25 % NaOcl was used for chemical disinfection. To minimize the variables between study groups, the final irrigation before root canal obturation was performed using saline because sodium hypochlorite causes tooth discoloration when mixed with MTA cement [34], and chelating agents such as EDTA 17 % or Glyde File Prep was not used because it could negatively affect the bond strengths of MTA-dentin [35, 36]. The size of the apical preparation in secondary treatment was larger than that in primary treatment to ensure removal of the previous root canal filling.

Controlling the length of root canal obturation is important from both biological and legal standpoints [37, 38]. Every measure was taken in this study to confine the obturation material within the root canal; however, extruded obturation material was observed in 14 teeth, mainly teeth with a history of root canal treatment. Extrusion of obturation material may be explained by root resorption from chronic inflammation or alteration of the apical configuration from previous treatment attempts [39]. Nevertheless, complete periapical healing and complete resorption of extruded material was observed in 13 out of 14 teeth in the follow-up radiographs. Therefore, extrusion of some obturation material was an inevitable event, and communicating this to patients before the treatment may minimize legal disputes.

This study was limited to female patients because it was conducted at Girls University Campus and faced difficulties recruiting patients diagnosed with chronic apical abscess because patients and general practitioners tend to select extraction over endodontic treatment for teeth with chronic apical abscesses. Therefore, the eligible teeth were randomized into study blocks of 4 teeth (TCTC) to facilitate treatment of eligible teeth, rather than waiting for long period of time to collect the recommended sample size before the intervention began. Finally, the evaluation of periapical healing was limited to periapical radiographs, which are less sensitive to apical periodontitis compared with cone beam computed tomography [40]. Future studies should include larger sample size, long term recall period and 3D imaging modalities.

## Conclusions

The outcomes of single-visit endodontic treatment of mature teeth with chronic apical abscesses using MTA cement were better, but not statistically significant, compared to conventional treatment. Mineral trioxide aggregate cement mixed in a 0.26 WP ratio facilitated the clinical manipulation of MTA cement and compaction into the narrow and curved canals of mature teeth.

## Additional file

Additional file 1: Periapical radiographs of study sample. Preoperative, postoperative, and follow-up radiographs of MTA and gutta-percha treated teeth. (PDF 583 kb)

## Abbreviations

MTA: Mineral trioxide aggregate
WP: Water to powder ratio
