# Supplementary material for: Single-visit endodontic treatment of mature teeth with chronic apical abscesses using mineral trioxide aggregate cement: a randomized clinical trial
Source: BMC Oral Health. 2016 Aug 23;16(1):78. doi: 10.1186/s12903-016-0276-y (PMC4994397; doi:10.1186/s12903-016-0276-y)
Supplement: Additional file 1: — Periapical radiographs of study sample. Preoperative, postoperative, and follow-up radiographs of MTA and gutta-percha treated teeth. (PDF 583 kb) [file 12903_2016_276_MOESM1_ESM.pdf]

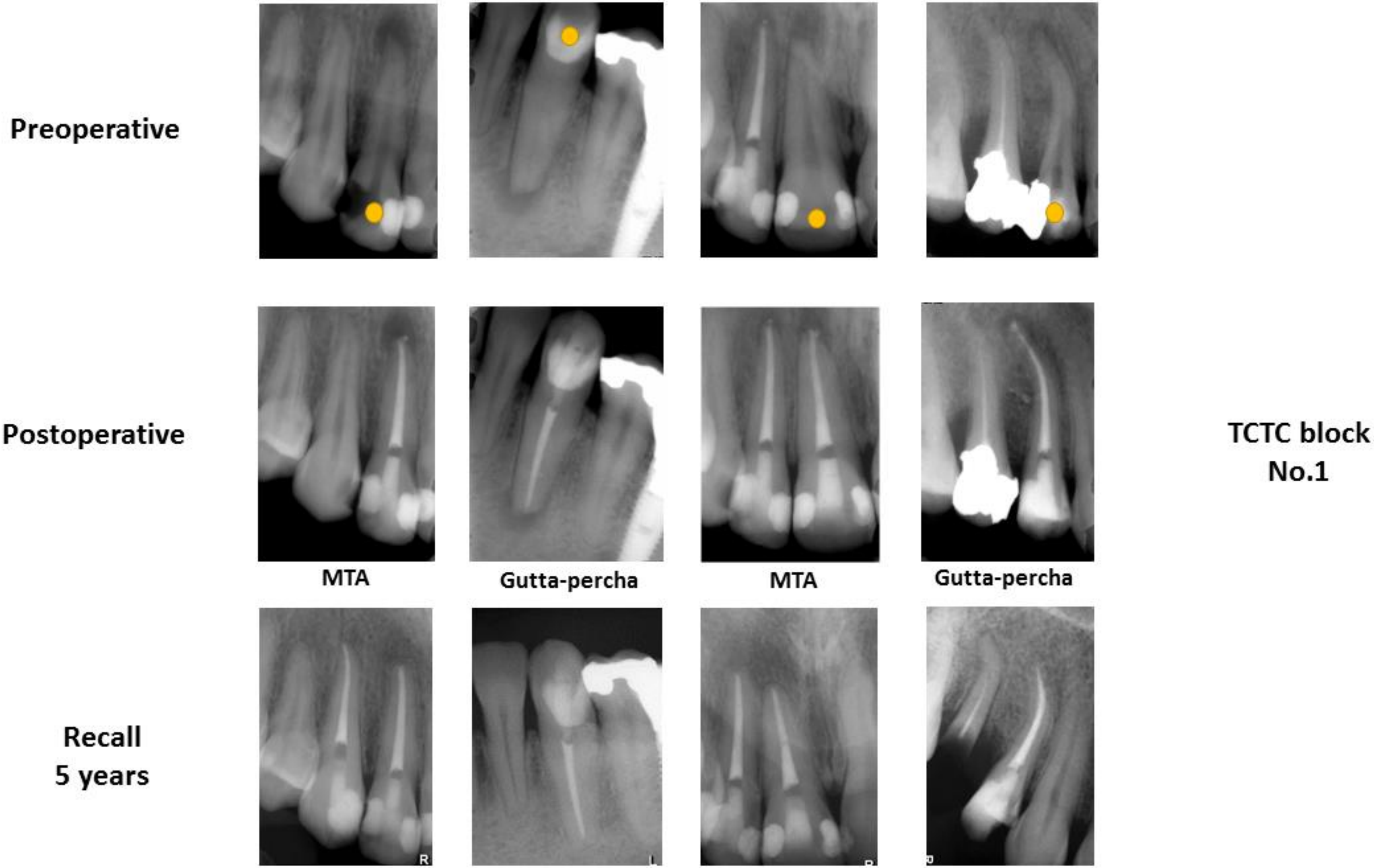

Copyright to R. Alsulaimani, College of Dentistry, King Saud University, Saudi Arabia

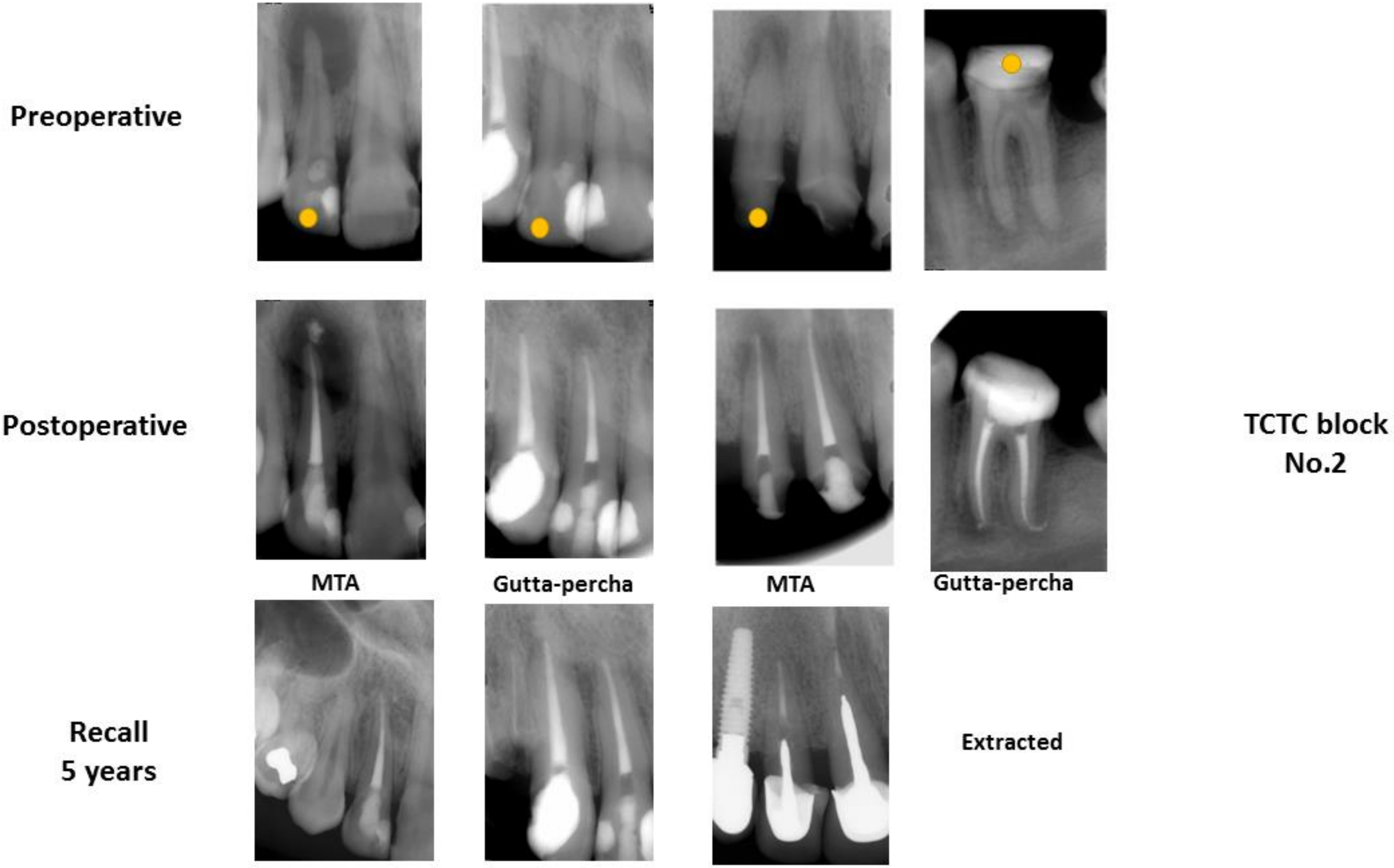

Copyright to R. Alsulaimani, College of Dentistry, King Saud University, Saudi Arabia

TCTC block No.3

Preoperative

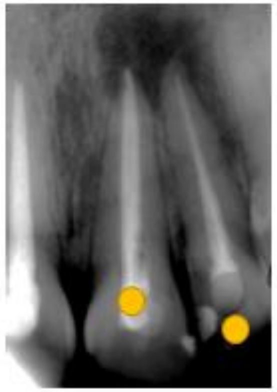

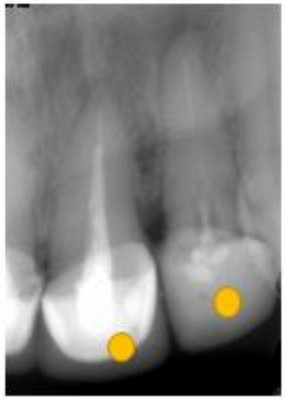

Postoperative

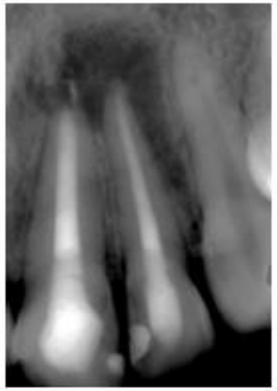

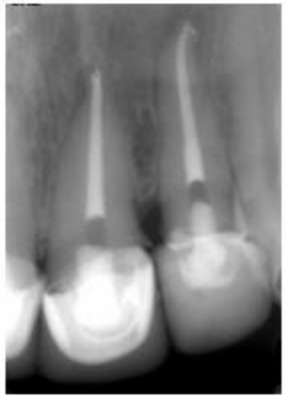

MTA

Gutta-percha

Recall
5 years

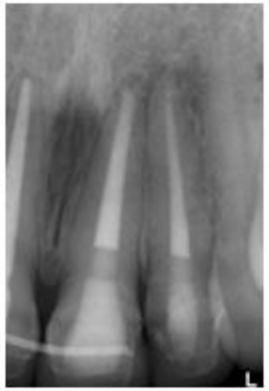

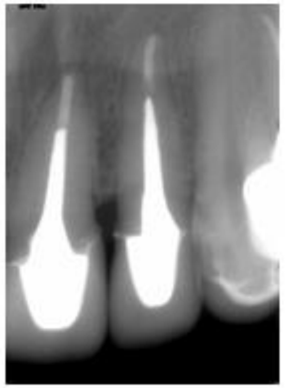

Copyright to R. Alsulaimani, College of Dentistry, King Saud University, Saudi Arabia

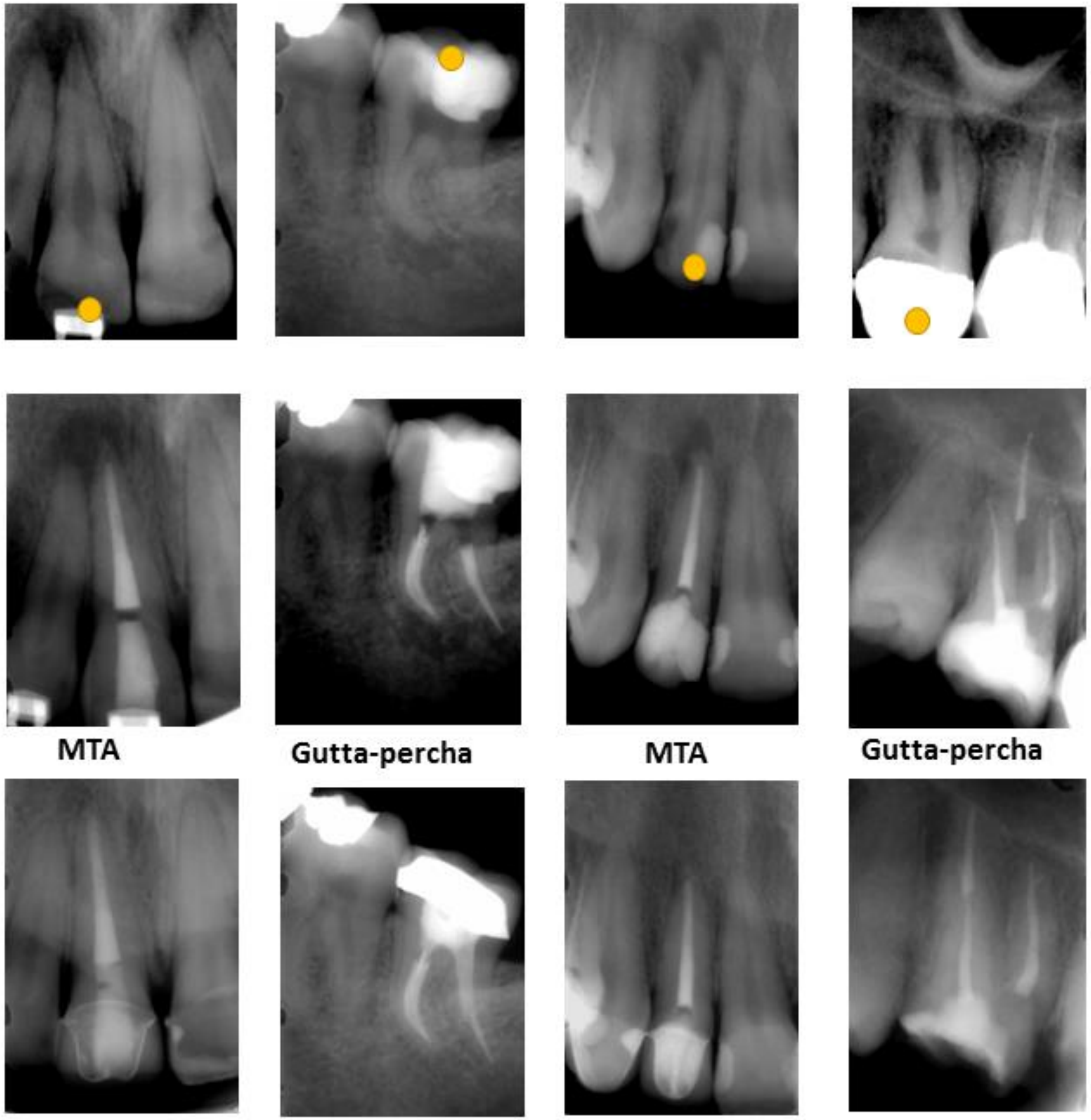

Copyright to R. Alsulaimani, College of Dentistry, King Saud University, Saudi Arabia

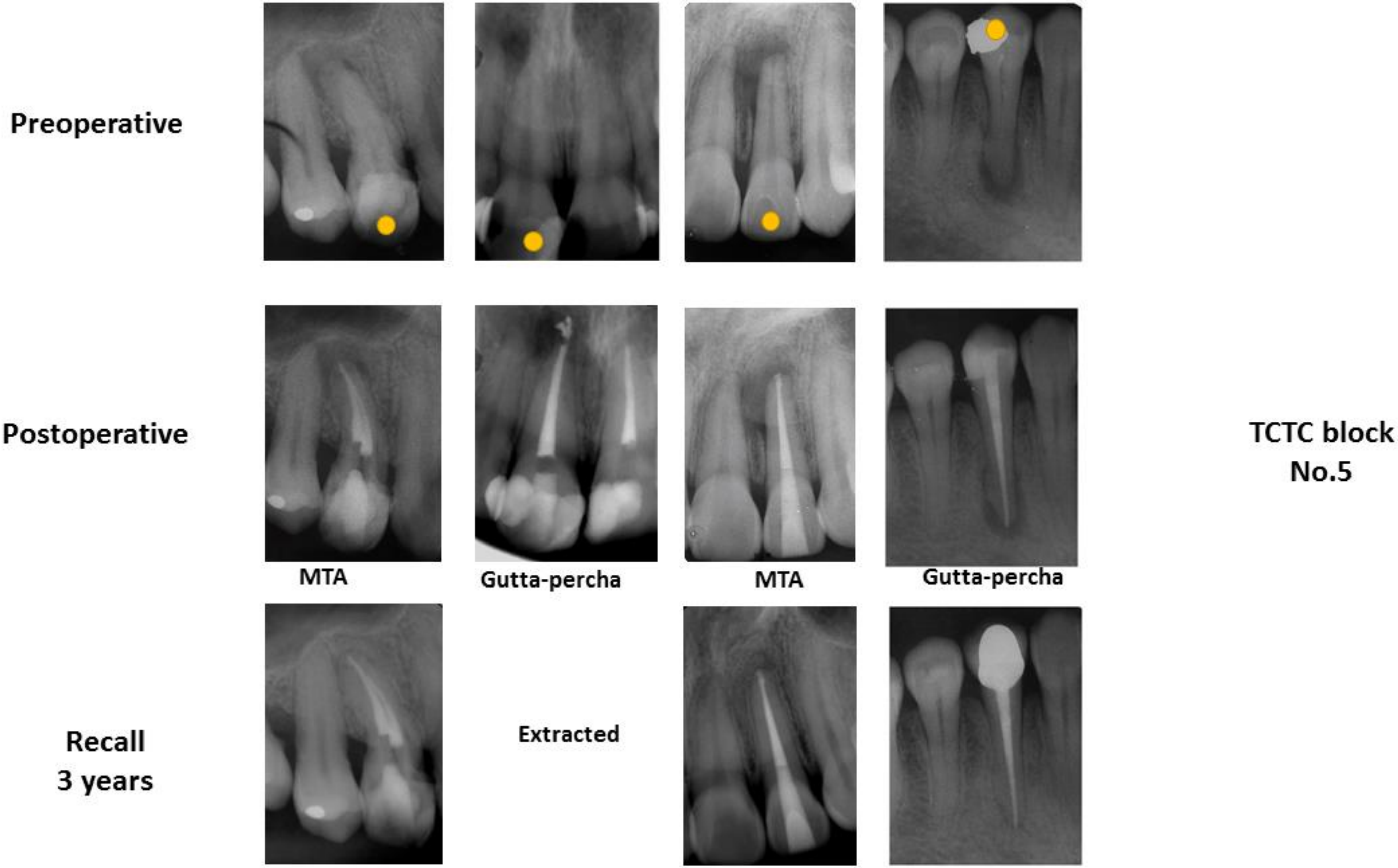

Copyright to R. Alsulaimani, College of Dentistry, King Saud University, Saudi Arabia

TCTC block No.6

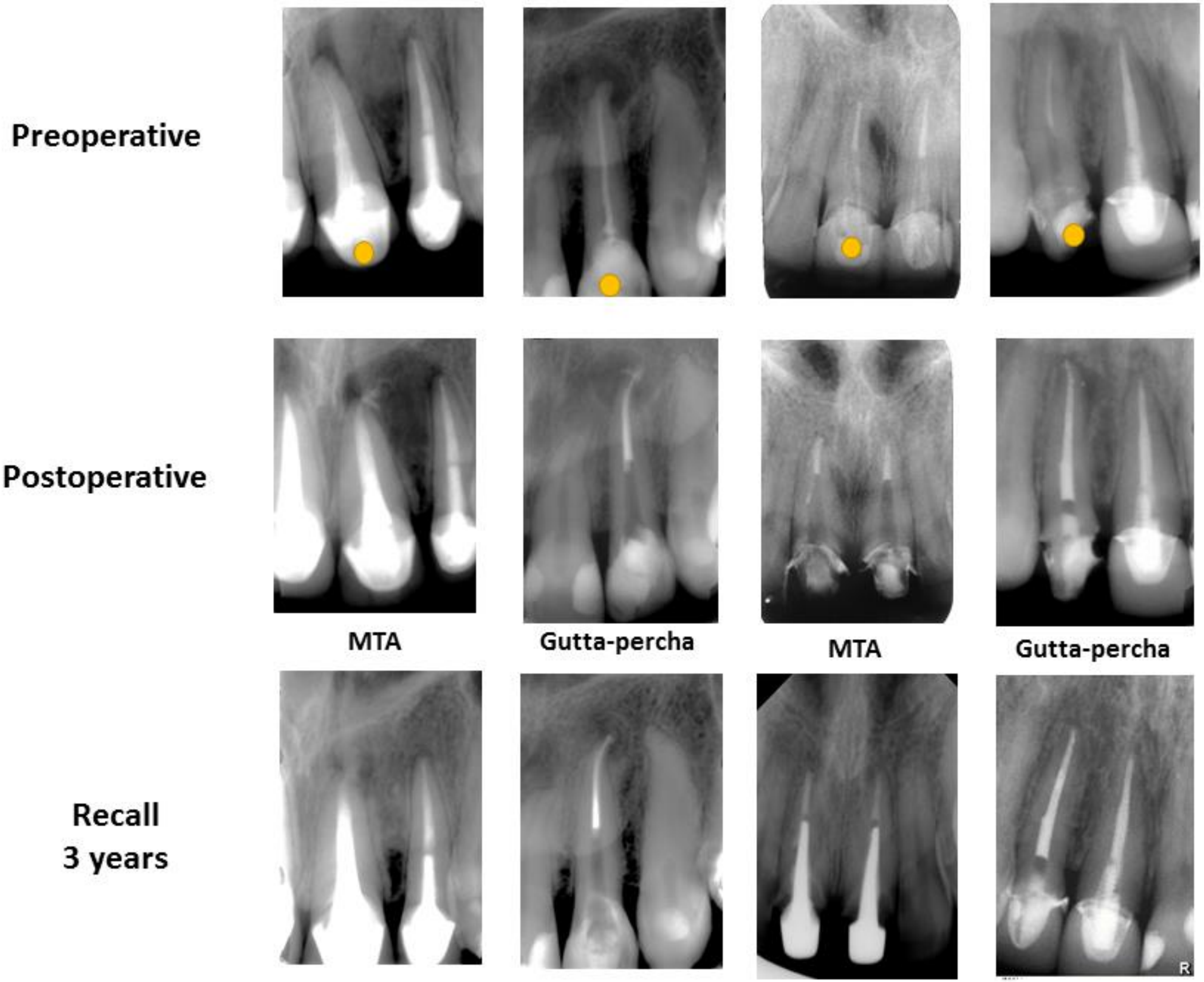

Copyright to R. Alsulaimani, College of Dentistry, King Saud University, Saudi Arabia

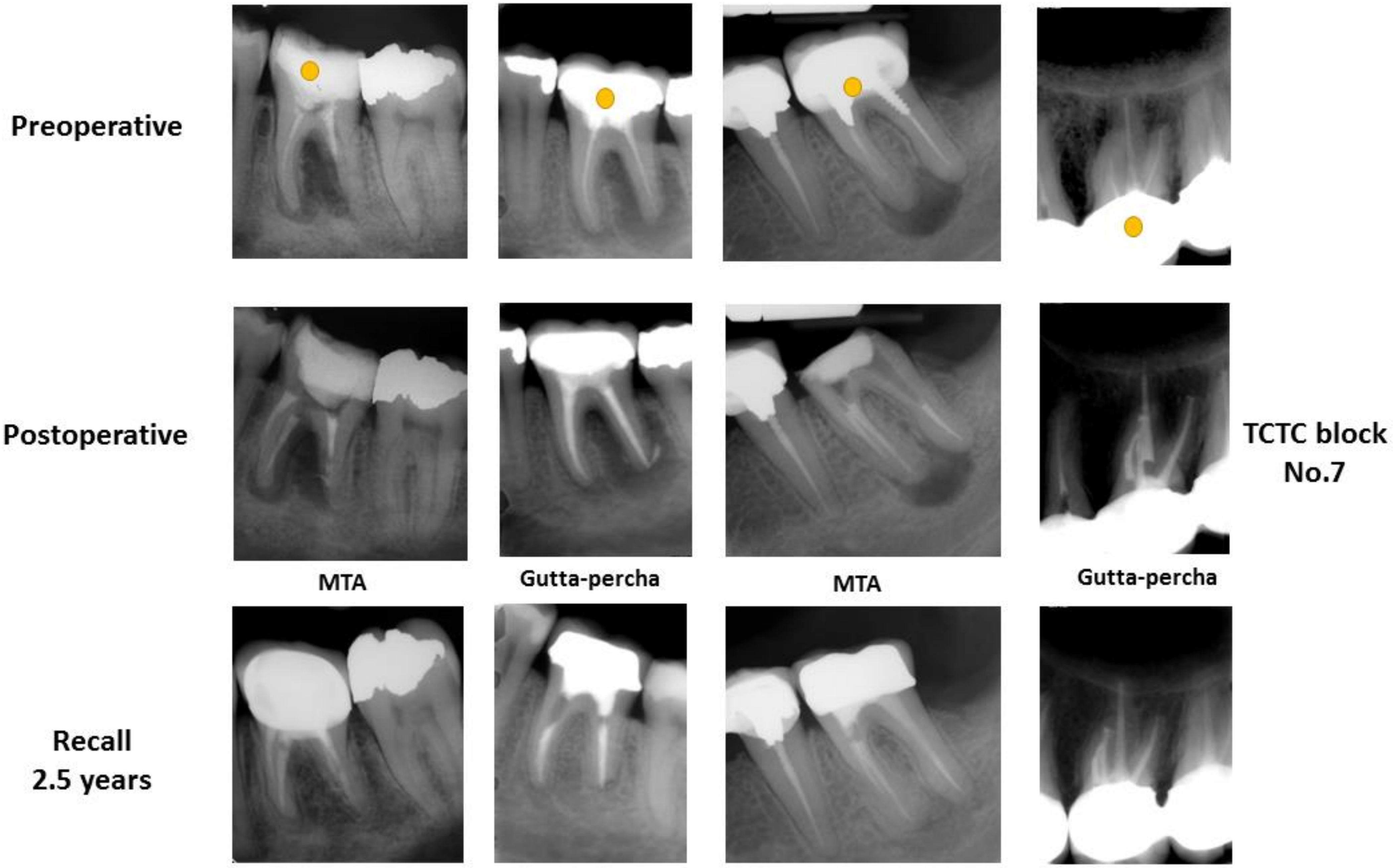

Copyright to R. Alsulaimani, College of Dentistry, King Saud University, Saudi Arabia

TCTC block
No.8

Preoperative

Postoperative

Recall
2.5 years

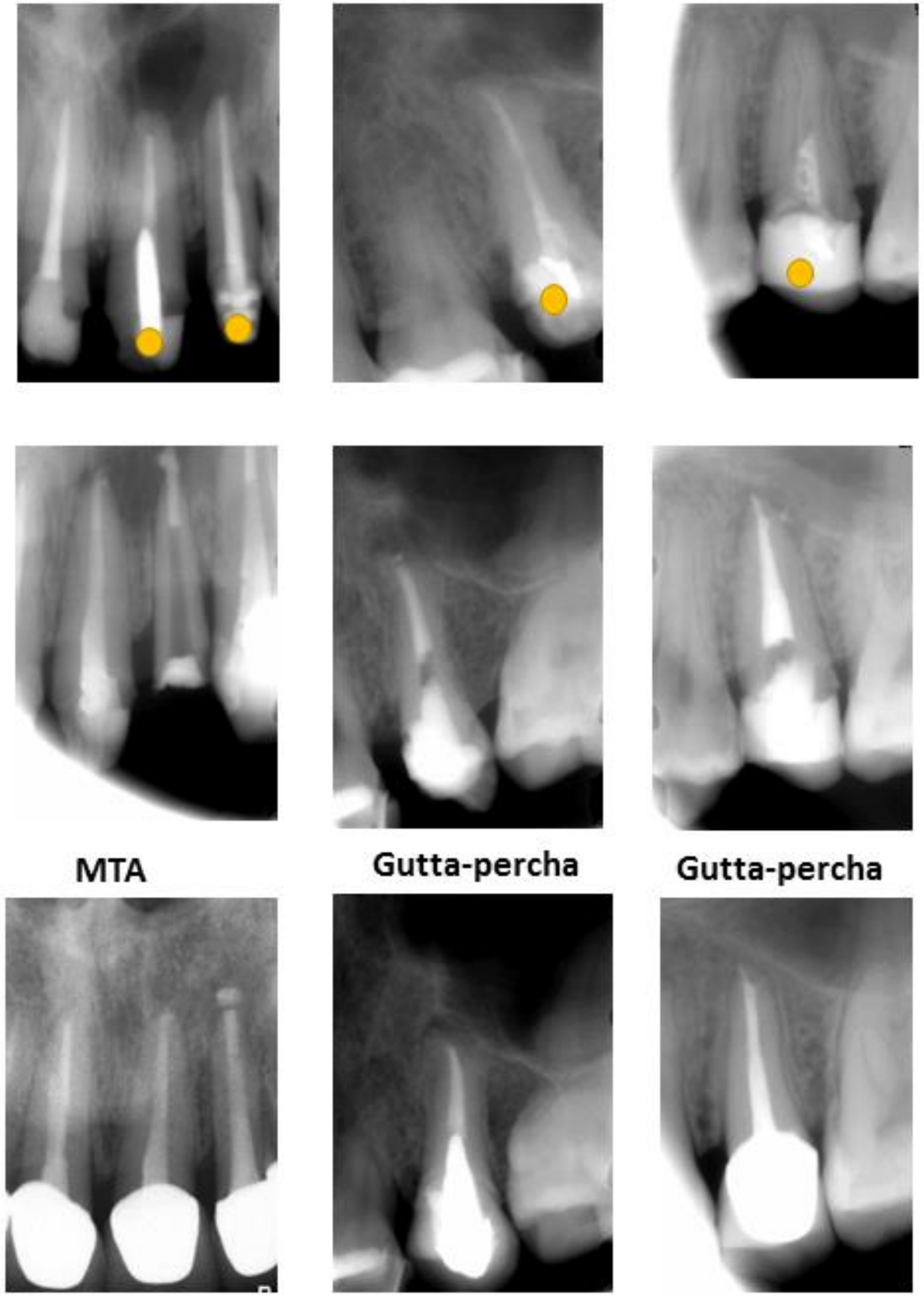

Copyright to R. Alsulaimani, College of Dentistry, King Saud University, Saudi Arabia
